# Speech air flow with and without face masks

**DOI:** 10.1038/s41598-021-04745-z

**Published:** 2022-01-17

**Authors:** Donald Derrick, Natalia Kabaliuk, Luke Longworth, Peiman Pishyar-Dehkordi, Mark Jermy

**Affiliations:** 1 New Zealand Institute of Language, Brain, and Behaviour, University of Canterbury, Christchurch, 8041 New Zealand; 2 Department of Mechanical Engineering, University of Canterbury, Christchurch, 8041 New Zealand; 3 Department of Linguistics, University of Canterbury, Christchurch, 8041 New Zealand

**Keywords:** Risk factors, Mechanical engineering, Public health, Fluid dynamics, Acoustics

## Abstract

Face masks slow exhaled air flow and sequester exhaled particles. There are many types of face masks on the market today, each having widely varying fits, filtering, and air redirection characteristics. While particle filtration and flow resistance from masks has been well studied, their effects on speech air flow has not. We built a schlieren system and recorded speech air flow with 14 different face masks, comparing it to mask-less speech. All of the face masks reduced air flow from speech, but some allowed air flow features to reach further than 40 cm from a speaker’s lips and nose within a few seconds, and all the face masks allowed some air to escape above the nose. Evidence from available literature shows that distancing and ventilation in higher-risk indoor environment provide more benefit than wearing a face mask. Our own research shows all the masks we tested provide some additional benefit of restricting air flow from a speaker. However, well-fitted mask specifically designed for the purpose of preventing the spread of disease reduce air flow the most. Future research will study the effects of face masks on speech communication in order to facilitate cost/benefit analysis of mask usage in various environments.

## Introduction

The earliest modern-era face masks were made of gauze^1^, and were used in conjunction with outdoor-air treatments for containment of the Spanish influenza pandemic of 1918^2^. Kellogg^3^ studied the efficacy of the face mask material, revealing that the gauze mesh allowed bacteria through the mask. While multiple layers or tighter gauze weave reduced the passage of bacteria through the mesh, the extra thickness also made breathing difficult and deformed the mask such that bacteria could travel through the sides of a mask^3^.

Nevertheless, face mask technology has both changed and proliferated since the gauze and cloth masks used during the pandemic of 1918. This proliferation of face masks is especially relevant dating from April 3, 2020, when the CDC changed initially skeptical guidelines to recommend masks, allowing a range of masks including (1) surgical masks, (2) masks that fit properly and are made of tightly-woven breathable fabric like cotton, with 2 or 3 layers or an inner filter pocket^4^. This change in guidelines, combined with the rapid increase in COVID-19 cases dating from mid-March, led to a worldwide commercial response resulting in a wide range of readily accessible pollen, cloth, surgical, and dust masks–so many that there have been considerable environmental roll-on effects as a result^5^.

On the lightest end of that spectrum are pollen-masks (polyethylene foam masks) designed only to filter out pollen particles (10–200 $\upmu$m) about two-three orders of magnitude larger than SARS-CoV-2 virion (100 nm^6^). Even though pollen tends to get through masks, masks reduce the pollen invasion rate by slowing pollen down and letting it deposit on the mucosa in the nasal passages and outer conjunctiva of the eyes instead of moving deeper^7^. Since pollen exposure is positively correlated with SARS-CoV-2 infection rates^8^, presumably wearing masks during pollen season reduces the risk of catching COVID-19 simply by reducing pollen exposure.

Next are the many home-made cloth face masks that vary greatly in construction, and as a result are impossible to assess as a whole^9^. However, cloth face masks can be made to block more air flow through the use of filters. The combined electrostatic action of the surface charge on electret fabrics, and filtering action of the two fabrics increase filtration to 80% of small particles (aerosols) and 90% of larger particles^10^. However, thicker layers make fabric stiffer and encourage mask leakage–especially at the upper edge beside the bridge of the nose and past the eyes^11^. A qualitative systematic review of recent studies has shown that cloth face masks have minimal impact on virus escape during normal breathing, and should only be used for a short period of time in indoor spaces with poor ventilation–where the risk of having no mask is greatest–when no other options such as improving ventilation exist^12^.

Surgical masks, today often made of three layers of non-woven (spunbound) polypropylene, were largely devised to prevent larger droplets from being projected during coughing, sneezing, speaking, and breathing^13^. The material itself is tested for bacterial, blood, and sub-micron (large virus size) particle filtering^13^. However, surgical masks were not designed to have a tight fit around the nose and sides of the face - they are loose-fitting masks.

Instead, the United States National Institute for Occupational Safety and Health (NIOSH) devised the N95 masks standard to provide greater protection. These masks are intended to have a tighter fit and greater filtration capacity in order to remove more blood and at least 95% of the particles that are hardest to remove, which are usually 0.1–0.3 $\upmu$m particles, both from and to the wearer^14^. Other countries have similar standards, like the People’s Republic of China’s KN95 standard, and the Korean K94 standard. The tight fit intended with these standards is very important–potentially infectious particles escape primarily through the imperfect face-seals, so the quality of the filter is not enough^15^.

Specific studies do show that mask material can filter bacteriophage MS2^16^ and virus particles produced by people with influenza^17^, and that N95 plus eye protection is better at preventing influenza infections of those exposed to same^18^ compared to not wearing eye protection or even just using a surgical mask. However, Nanda et al’s meta-analysis shows that most studies of mask efficacy (for all mask types) are of limited quality and not SARS-CoV-2 specific^19^. They found no conclusive evidence that surgical face masks effectively stop the spread of respiratory viruses^19^. Bartoszko et al. found a similar lack of results in their systematic review published at the beginning of the COVID-19 pandemic^20^.

Research into face masks generally shows trade-offs between the three choices of: (1) masks that leak particles due to the use of porous materials; (2) masks that leak fewer particles, but by added rigidity or required respiratory force deform and allow leaks out the sides, or (3) masks that do neither but are then expensive or uncomfortable enough the public does not readily comply with their use. These three themes have remained apparent throughout the entire history of the literature on the filtering capacity of face mask materials.

Due largely to the differences in mask fit on inhalation and exhalation, the degree of inward protection (reducing the number of infectious particles inhaled by the mask wearer) may be very different from the outward protection (reducing the number of particles directed towards persons near an infectious mask wearer)^21^.

The resulting frustration amongst scientists regarding the effectiveness of face masks has inspired generations of mask use skepticism starting from the flu pandemic of 1918. Kellogg concluded at the time that the use of gauze masks only slowed the rate of infection by at most 50%, and so was not warranted as a compulsory public measure for slowing the spread of epidemics^3^. In more recent times, in a large-scale study of nurses in Ontario, real-world influenza infection rates were 22–23% during the 2008–2009 flu season, and equivalent regardless of mask-type used^22^. In 2021, the lack of real-world evidence for the efficacy of masks in preventing respiratory infection has influenced at least one doctor to publish the opinion that more research needs to be done for mask usage recommendations to meet AGREE II medical guidelines^23^.

Nevertheless, the evidence is not all bad news for face mask use. All mask materials do capture at least some SARS-CoV-2 particles, from a low of 20–60% filtration efficiency for home-made masks, to a high of 98% for N95 mask materials^24^ when properly fitted. In particular, masks–especially surgical masks–slow forward spread of particles of all sizes^25^, reducing the exposure of persons that the wearer is facing.

The most recent models and analysis show that since face masks can filter some particles, they can all reduce the likelihood of spread of SARS-CoV-2 in specific situations. It must be noted that even experts can fit masks badly, and this greatly reduces their real-world effectiveness^26^. However, the following trends still apply: In extremely low viral load environments, such as the outdoors in uncrowded areas, masks are highly unlikely to be necessary. In low virus load environments, moderately effective filtering from cloth or surgical masks will reduce the likelihood of infection spread, and as virus load increases, higher levels of protection, such as N95 masks, eye protection (or even full coveralls) are needed to be effective^27^. The research shows that the advantage of masks is largely that of slowing disease spread, not preventing it. However, the slow-down generated by both higher face mask use in indoor situations, along with higher rates of social distancing, can not only flatten the curve, but bring the R-naught of SARS-CoV-2 below 1, effectively controlling disease spread^28^.

Given these results, the most beneficial space for continued face mask research is in charting air flow transmission of people wearing various types of face masks, to inform on the best choices of types of face masks. To accomplish this task, we used schlieren imaging to capture the large air flow patterns produced by speakers wearing a range of face masks. The method captures eddies down to 487 $\upmu$m in size. An area in front of the speaker’s face, extending across the 400 mm span of the mirror, is captured. Schlieren imaging visualizes changes in air refractive index, i.e. is primarily a measurement of changes in air temperature. It thus visualizes the air flow associated with the warm air exhaled during speech. The method does not measure the concentration of potentially infectious particles and we do not comment on the effect of masks on infection.

Schlieren imaging has been used in mask research before: Tang et al.^29^ obtained high speed schlieren images of people coughing with and without surgical and N95 masks, demonstrating that the turbulent jet produced by an open mouth cough is redirected by a surgical mask and blocked by an N95 mask. Coughs flows leak out the top, bottom, sides, and even the front of masks. Coughs involve air flow up to 8 m/s^29^ [see Fig. 4]. The speed and pressure of coughing exceed that caused by speech. Other workers have studied the effect of masks on coughing^30,31^, coughing and breathing^32,33^ and breathing^34^. There are studies which image airflow produced during speech^33,35^, but none with a mask. The effect of masks on speech acoustics has been studied^36–38^, but these studies did not measure air flow. This study is the first to image air flow produced by speech with masks.

### Goals and hypotheses

The goal of this research is to survey many masks to give the scientific community and public an idea of how various types of masks control air flow from a speaker. Based on the literature, we have these hypotheses:

1. All masks will slow down air flow compared to speech without an air mask.
2. Masks that are porous enough will allow air flow out the front, but attenuate its speed and distance of motion.
3. Masks that are less porous will stop air flow out the front, but allow air flow to leak out the top, bottom, and sides.
  - Masks with a closer fit will allow this flow to occur slowly and continuously
  - Masks with poor fit will have areas of relatively unimpeded air flow at the site of the large leak.

## Methods

This research focused on studying large eddy formation from speech air flow during speech with and without face masks.

### Declaration

The University of Canterbury’s Human Ethics Committee (HEC) approved ethics for this study (HEC 2020/43). The experiment was performed in accordance with the relevant named guidelines, regulations, and agreed-upon procedures listed in the HEC 2020/43 document.

### Participants

We recorded one participant, a 23 year-old male with facial hair. The speaker was a native speaker of New Zealand English with intermediate knowledge of te reo Māori. The participant reported no speech or hearing issues.

### Setup and materials

**Figure 1:**
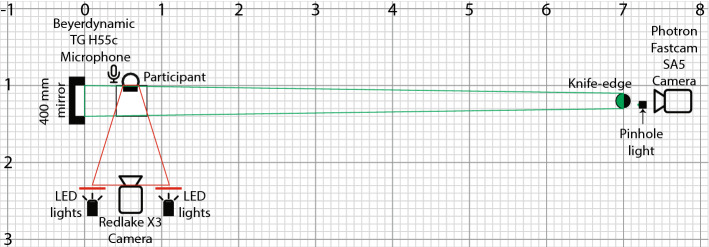
Schematic of schlieren and video setup, to scale in a 0.1 m grid.

The experimental setup included a single-mirror schlieren system for air flow visualization, a system for videoing face motion, and a head-mounted microphone system for speech recording. A table and chair was set up so the speaker sat with the mirror adjacent to the right of their head. The face motion camera was set across from the speaker to capture facial motion along the coronal plane. The schlieren motion camera was placed in front of the mirror 7 m away so that the speaker’s head was captured along the sagittal plane with the face pointing left. The equipment positioning is shown in Fig. 1.

**Figure 2:**
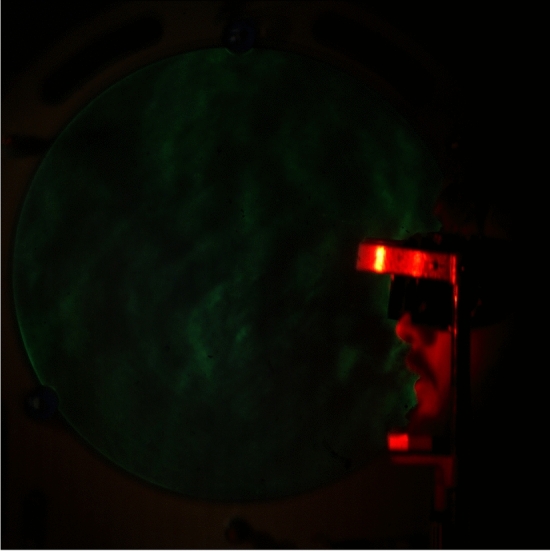
Video frame of Schlieren imaging—frame 2001, no-mask image, first phrase.

**Figure 3:**
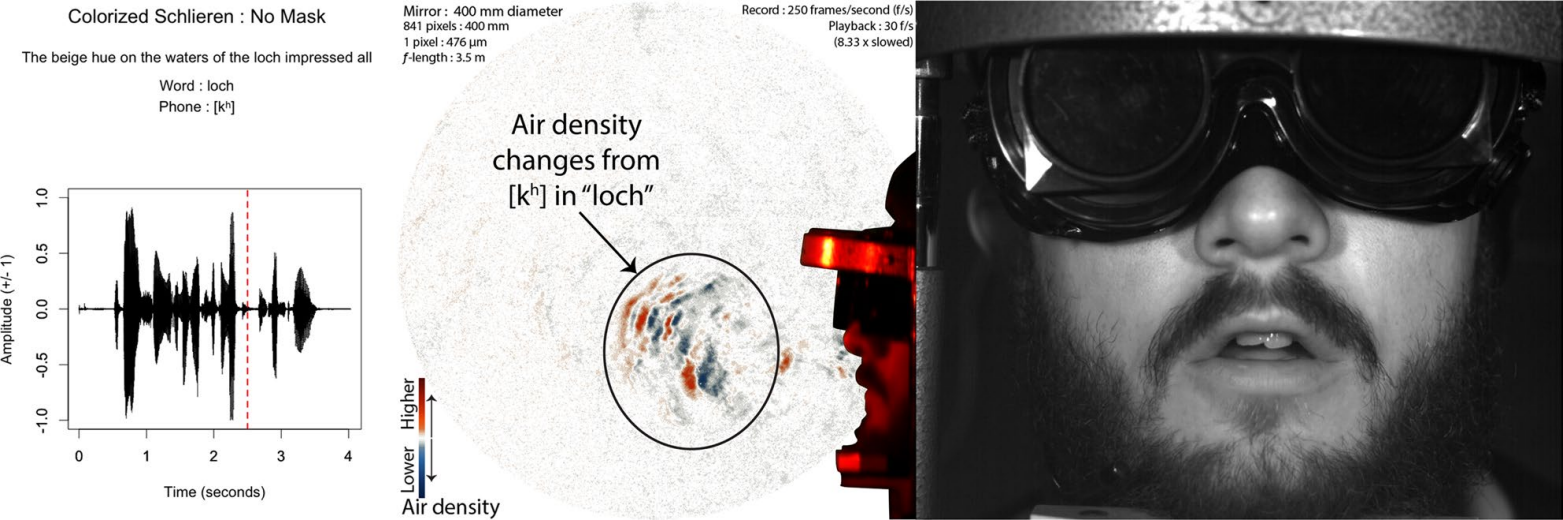
Audio, Schlieren, and Video of speech without a face mask (Frame 625, 1st block, no face mask). Image from 73 milliseconds (ms) after the lip opening and release burst for the [k$^{h}$] in “loch”. Note that the k’s puff is strong and well-defined, with eddies that change air-density across the span of the puff. The red-dashed line in the audio waveform indicates the timing of the video and schlieren frames used in this image.

The single-mirror schlieren system consisted of a green LED light source with a pinhole (Edmund Optics Inc., Barrington, NJ, USA), and a 400 mm diameter, 3.5 m focal length parabolic mirror, a knife edge on a linear stage with a micrometer adjustment, an 18–400 mm f/3.5–6.3 zoom lens (Tamron HB028, Saitama City, Japan) and a Photron SA5 camera (Photron, Tokyo, Japan, RGB, 1024 $\times$ 1024 px, 250 fps), as seen in Fig. 2. The resulting spatial resolution was 476 microns ($\upmu$m) per pixel. The field of view of the schlieren images was 400 mm and included the complete outline of participant’s face, as seen in Fig. 3. Schlieren recordings included a blue LED light (10–12 mA) mounted just above the mirror. The light was connected to an Arduino Uno circuit that converted audio amplitude to light intensity to allow audio/schlieren alignment in post-processing. Both the mirror and the Photron camera were levelled and positioned on optical benches with tripod mounts. The Photron’s bench had a linear positioning stage for adjustment along the line of sight.

The participant’s face was illuminated by two 10,000 lumen LED lights (CREE Inc., Durham, NC, USA) in a custom housing with parabolic reflectors and imaged with a standard 55 mm focal length Nikkor lens (Nikon, Tokyo, Japan) and MotionPro X3 camera (Redlake Inc., Tucson, AZ, USA, monochrome, 1280 $\times$ 1024 px, 250 fps). The spatial resolution of the facial motion images was 176 $\upmu$m per pixel.

Audio recording of speech used a condenser microphone headset (Beyerdynamic TG H55c) attached to a USB PRE 2 microphone phantom power pre-amplifier. The audio and video systems were connected to the same Windows machine (Windows 10, 16 GB memory, 1 GB SSD). Audio was recorded using Audacity (Audacity team, 3.0.2). Schlieren video was recorded using the Photron FASTCAM viewer software (PFV 4). Facial video was recorded using the Redlake MotionPRO X viewer software. The flow of the experiment was controlled using custom software written in LabVIEW (NI, Austin, Texas, USA).

Stimuli included the English sentence: “1: The beige hues on the waters of the loch impressed all 2: Including the French queen 3: before she heard the symphony again 4: Just as young prince Arthur wanted”. The phrase was divided into four parts, as indicated by the numbering. This sentence is a pangram, containing all of the individual phones of English. The sentence therefore provides examples of the full range of turbulent and laminar air flow patterns used in English. In so doing, the stimuli provides the appropriate ‘stress test’ of conversation speech on face mask performance.

The experiment included 13 different face masks in sequence (one of the masks was used twice in sequence, with and without the filter insert). Mask standards tested included KF94 (2), PM2.5 (3), KN95 (4, 11), PM2.5 (5, 12), VFE 98% (7), DET30(9), level 1 surgical (10, 13, 15), and unknown standards (6, 14). Mask types included surgical masks (2, 5, 10, 13, 15), multi-use cloth masks (3, 4, 6, 7, 9, 14), a carbon mask (8), a dust mask (11), and a pollen mask (12). Masks were manufactured in Korea (2, 11), Singapore (3, 5, 6, 7, 9, 10, 12), China (4), Japan (5), Thailand (8), New Zealand (13, 14), and Bangladesh (15). Materials used included polypropylene (2, 5, 10, 11, 13, 15), cotton (3, 14), unknown cloth (4, 6, 7, 8, 9), and polyurethane (12). The list of masks and their details can be found in Table 1.

**Table 1:** List of face masks used.

Number	Brand name	Manufacturer	URL	Materials	Size	Standard	Uses	Origin
2	KF94	Onnuriplan	Onnuriplan	3-ply polypropylene	large	KF94	once	Korea
3	Anti-bacterial face mask	UltraMask	Ultramask	3-Ply anti-bacterial cotton	One	PM2.5	Multiple	Singapore
4	Smart anti-bacterial protective mask	Pycom	Obsolete	Anti-bacterial knitwear	One	KN95	Multiple	China
5	Unicharm	Unicharm	Out of business	3-Ply polypropalene	One	PM2.5	Once	Japan
6	Decks Maskfit	Temasek	Temasek	Anti-bacterial cloth	XL	NO FILTER	Multiple	Singapore
7	Decks Maskfit	Temasek	Temasek	Anti-bacterial cloth + filter	XL	VFE 98%	Multiple	Singapore
8	Advantage carbon face mask	Healthy+	Out of business	6-Ply non-woven fabric	One	Charcoal	Once	Thailand
9	Temasek silk	Temasek	Obsolete	Satin cloth	One	DET30	Multiple	Singapore
10	Stylemaster	Stylemaster	Stylemaster	3-Ply woven polypropylene	One	Surgical	Once	Singapore
11	H910VPlus	Honeywell	Honeywell	Polypropylene fibre	One	KN95	Once	Korea
12	CORI Supermask	Cori	CORI	Microcell polyurethane foam	One	PM2.5 foam	Multiple	Singapore
13	Henry Schlein surgical mask (level 2)	Henry Schein	Henry Schlein	3-Ply polypropylene	One	Surgical	Once	New Zealand
14	Homemade cloth			Cotton	One	None	Multiple	New Zealand
15	Getwell	Getwell	Getwell	3-Ply polypropylene	One	Surgical	Once	Bangladesh

### Procedure

Written informed consent has been obtained from the participant under study. The participant agreed to the publication of their image in this online open-access scientific journal. The participant was seated at the table in Fig. 1, and given a pair of welding goggles (AS/NZS 1338.1 certified shade 5 filter goggles) to wear. The goggles provided protection against the two bright red-shaded lights used to illuminate the face of the speaker. The participant was also given the head-mounted microphone to wear.

The participant was instructed to place his forehead on the chin- and head-rest to stabilise the head and allow video recording of his facial motion during speech. The participant kept his chin above the chin-rest in order to allow free motion of his jaw. LabVIEW software was used to control the flow of data collection such that the participant would hear a recording of the phrase part that he was to repeat. The experiment controller then started recording with both cameras and the microphone, and then gave a verbal instruction to the participant to speak. The participant then spoke. This process was repeated for each of the four phrase parts, constituting one block of recording.

The experiment consisted of 15 blocks of recordings, The first block was recorded without a face mask, and then 14 more blocks with each face mask in the order listed in Table 1, such that each mask was used to record the English sentence once. We therefore collected 60 (15 $\times$ 4) recordings, and the process took approximately 6.5 hours, with one break for lunch and two 15-min coffee breaks.

### Data processing

The audio recordings were clipped to provide 500 ms of padding at the beginning and end of each recording. The audio recordings were then labelled and transcribed in PRAAT^39^ with narrow phonetic transcription. This provided direct information to relate speech sounds to any recorded air flow of varying density from the schlieren imaging.

A custom-made bash shell script running ffmpeg^40^ and imagemagick^41^ was then used to convert the black and green schlieren frames into a contrast-enhanced orange-blue diverging image^42^. For each frame from the second to the last frame, the preceding frame was converted to a black and white 8-bit negative, and then the current image was converted to a black and white 8-bit positive image. The frames were then overlaid 50% each, and light balanced so that they averaged 128 bit intensity. The black and white frame was then converted to an orange-blue diverging color image. The middle 5 intensities were then reduced to white in order to provide maximum contrast to make air density changes from air flow maximally visible to the human eye. This process produced a sequence of frames that showed air density changes over every 4 ms period of time during the audio recording.

A custom-made software written in R^43^ was then used to autocorrelate audio and schlieren files, and produced images of the audio waveform with indication of timing position. Each alignment was double-checked and adjusted as needed through visual analysis.

Another custom made bash script with ffmpeg and imagemagick was used to lay out the audio and schlieren images (for speech with face masks), and the audio, schlieren, and visual images (for speech without a face mask), along with word and phone labels, mask labels, and a schlieren scale label. This process allowed slide and video-based qualitative analysis of the resulting data.

## Results

**Figure 4:**
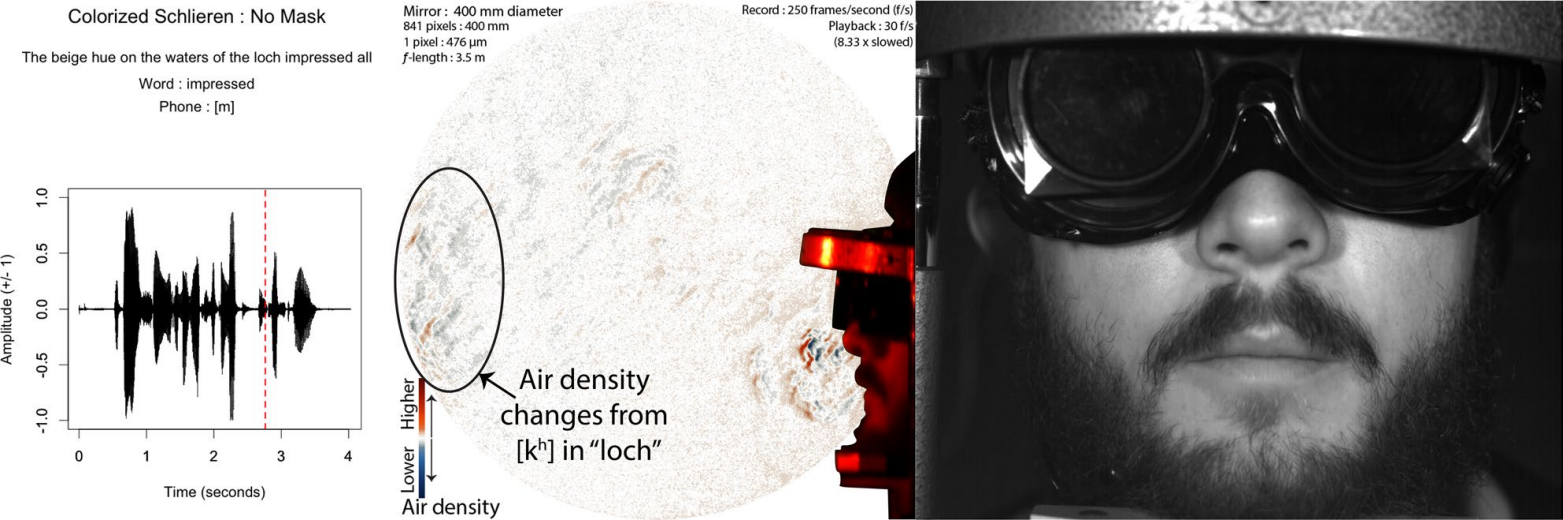
Audio, Schlieren, and Video of speech without a face mask (Frame 691, 1st block, no face mask). Image from 337 ms after the lip opening and release burst for the [k$^{h}$] in “loch”. The k’s puff spans the entire length of the mirror, past 35 cm of distance from the lips of the speaker. The red-dashed line in the audio waveform indicates the timing of the video and schlieren frames.

The results of this research now show that speech without any face mask allows clear patterns of air flow from the mouth and nose. Speech that is produced from quick releases of high pressure in the mouth, as in the release burst (lip opening releasing air pressure) and aspiration (extra forced air from the lungs) from the “k” ([k$^{\varvec{h}}$]) of the word “loch”, tend to produce the fastest moving air from speech. We show examples of this in Figs. 3 and 4. At about 73 ms, this air flow has spanned over 200 mm away from the lips, as seen in Fig. 3. The schlieren image preserves detailed information about the changes in air density related to this puff of air.

The air from the release continues to propel forward, such that by 337 ms, it has spanned across the 400 mm mirror into the room, as seen in Fig. 4.

**Figure 5:**
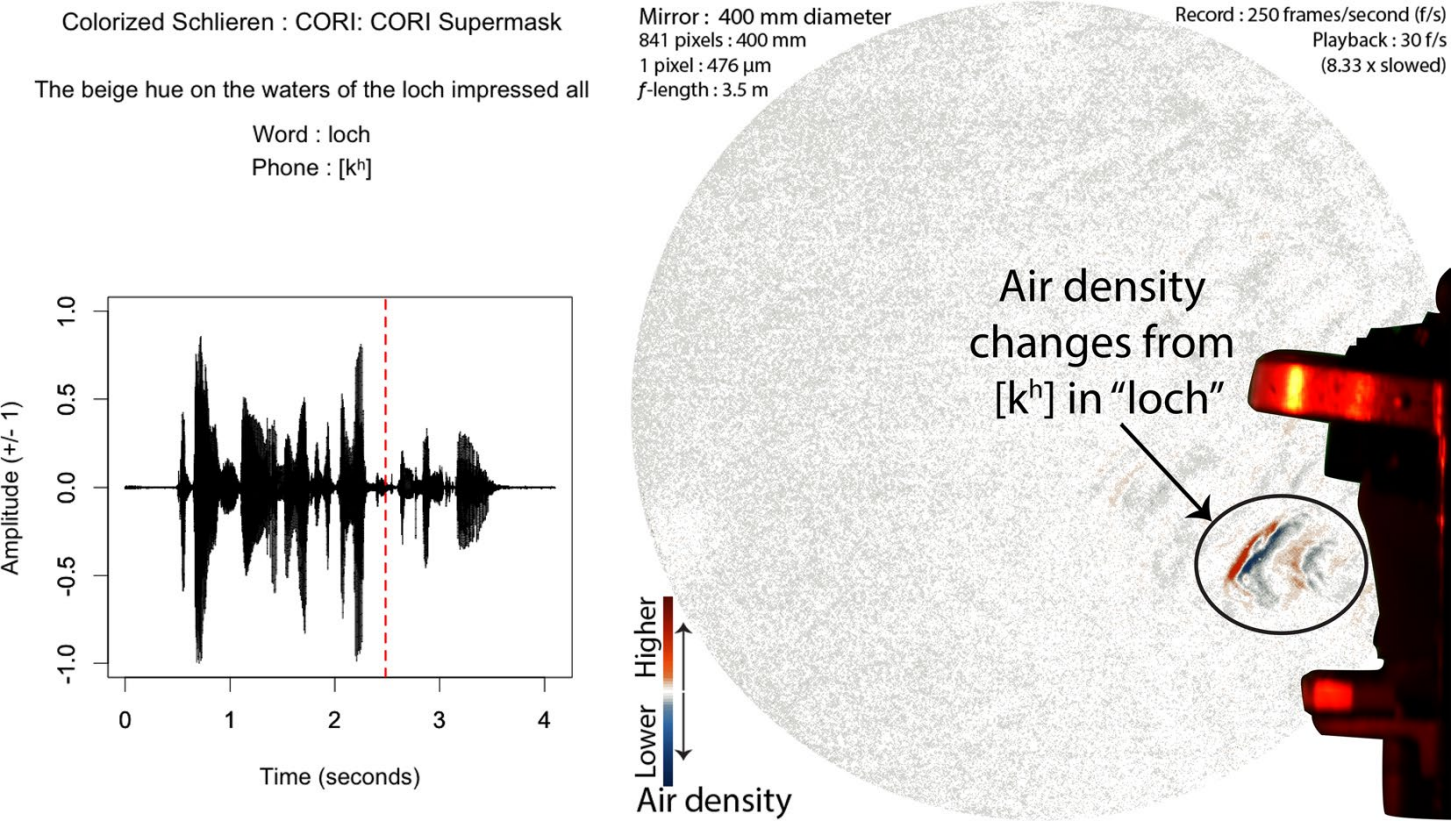
Audio and Schlieren of speech through a porous face mask (Frame 621, 1st block, CORI Supermask). Image from 88 ms after the release burst for the [k$^{h}$] in “loch”. Note that the k’s puff is smoother and less well-defined than the one in Fig. 2, but still has eddies that change air-density across the span of the puff. The red-dashed line in the audio waveform indicates the timing of the schlieren frame.

In contrast, all of the face masks alter air flow considerably in comparison to speech without a face mask. The most porous of the masks, the CORI Supermask, allows air flow trough the mask, as seen in Fig. 5. However, the release burst and aspiration from “loch” at 88 ms has travelled only about 10 cm compared to over 20 cm from Fig. 3. It also shows much clearer and stable patterns of high and low density changes compared to the air flow seen in Fig. 3.

**Figure 6:**
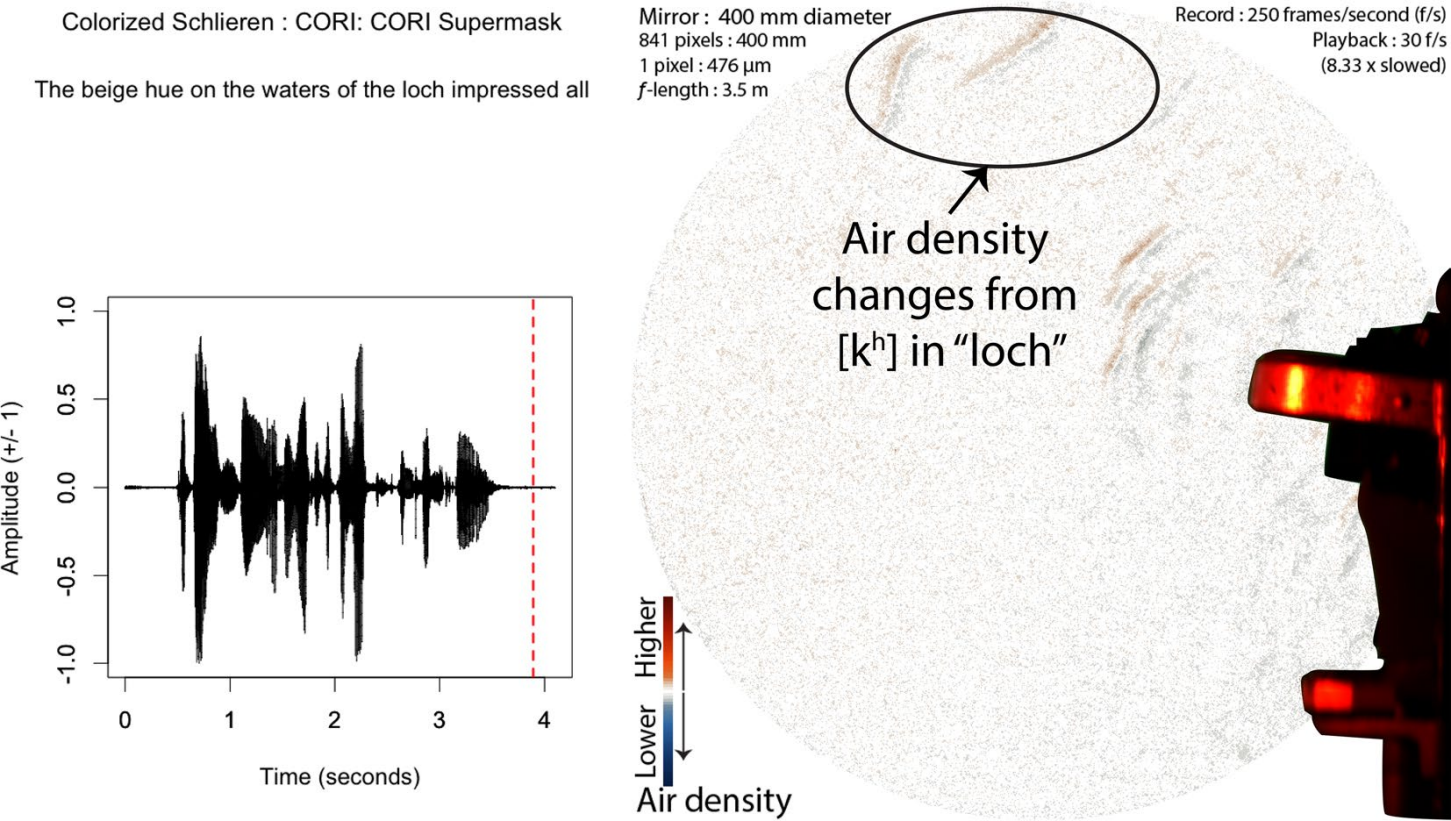
Audio and Schlieren of speech through a porous face mask (Frame 973, 1st block, CORI Supermask). Image from 1,496 ms after the release burst for the [k$^{h}$] in “loch”. Note that the k’s puff has moved slow enough that it floated up to the top of the mirror’s span, compared to nearly straight across as in Fig. 4. The red-dashed line in the audio waveform indicates the timing of the schlieren frame.

The release burst from the “k” does span the mirror, but because it moves so slowly, it takes about 1.5 s to get across the mirror, and instead of projecting forward, it slowly floats upwards as well as across, as seen in Fig. 6.

**Figure 7:**
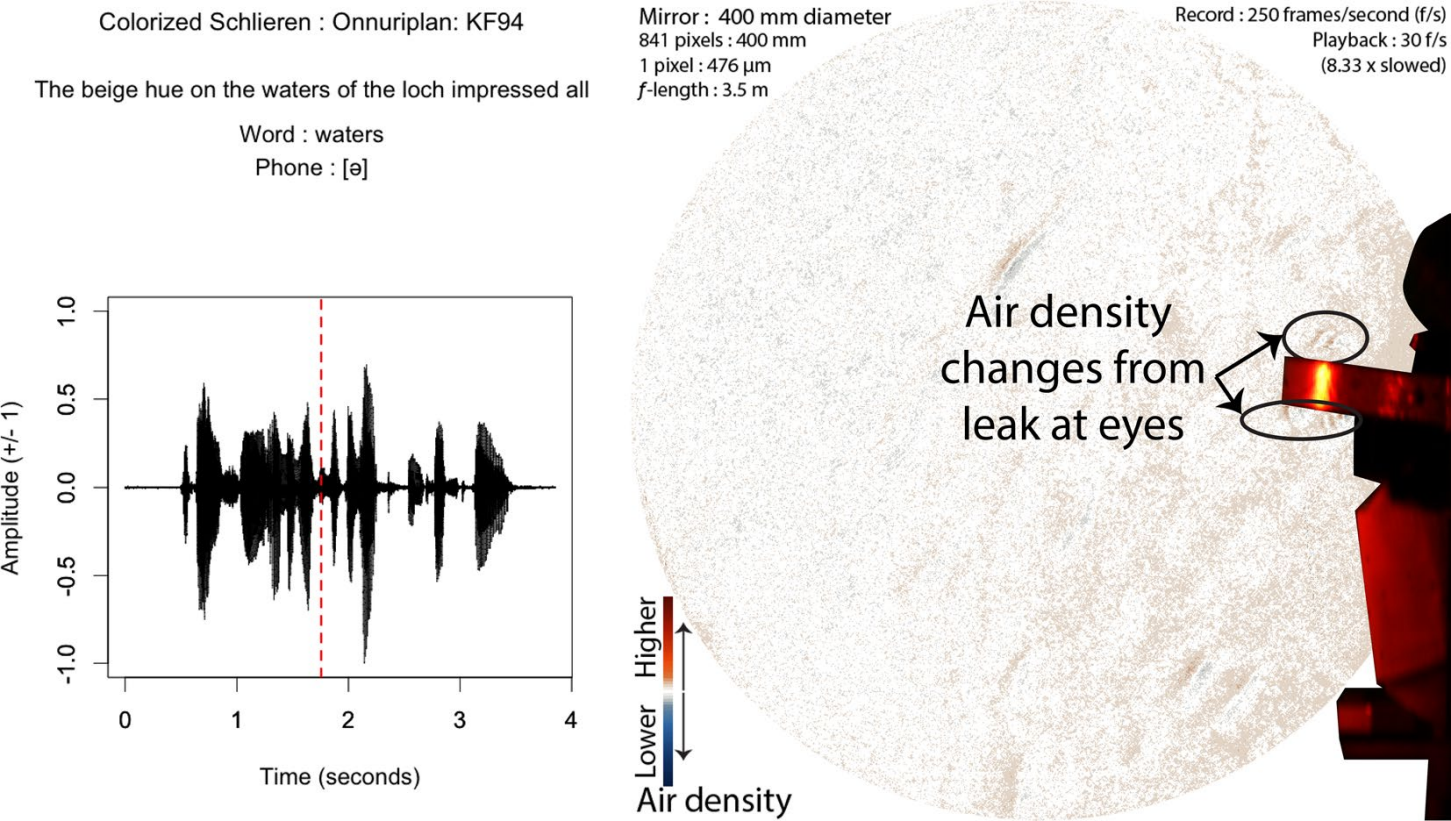
Audio and Schlieren of speech through a KN 94 mask with a slight leak (Frame 439, 1st block, Onnuriplan KN 94). The air flow comes out in front and above the eyes and below the chin. The air flow moves slowly forward and upwards. The red-dashed line in the audio waveform indicates the timing of the schlieren frame.

The other masks stop almost all of the forward momentum of air flow. Instead, air leaks out in one of two ways. If the mask does not fit well, it allows small leaks where speech air flow is expelled relatively quickly, forming evidence of clear leaks. We have very few cases of this type of leak, but one was nicely visible from the Onnuriplan KN 94 dust mask, as seen in Fig. 7. Leaks like this can be felt around the eyes, and so the participant inadvertently fixed this leak before completing the next three blocks. Eddies from around the eyes can be clearly seen in the image.

**Figure 8:**
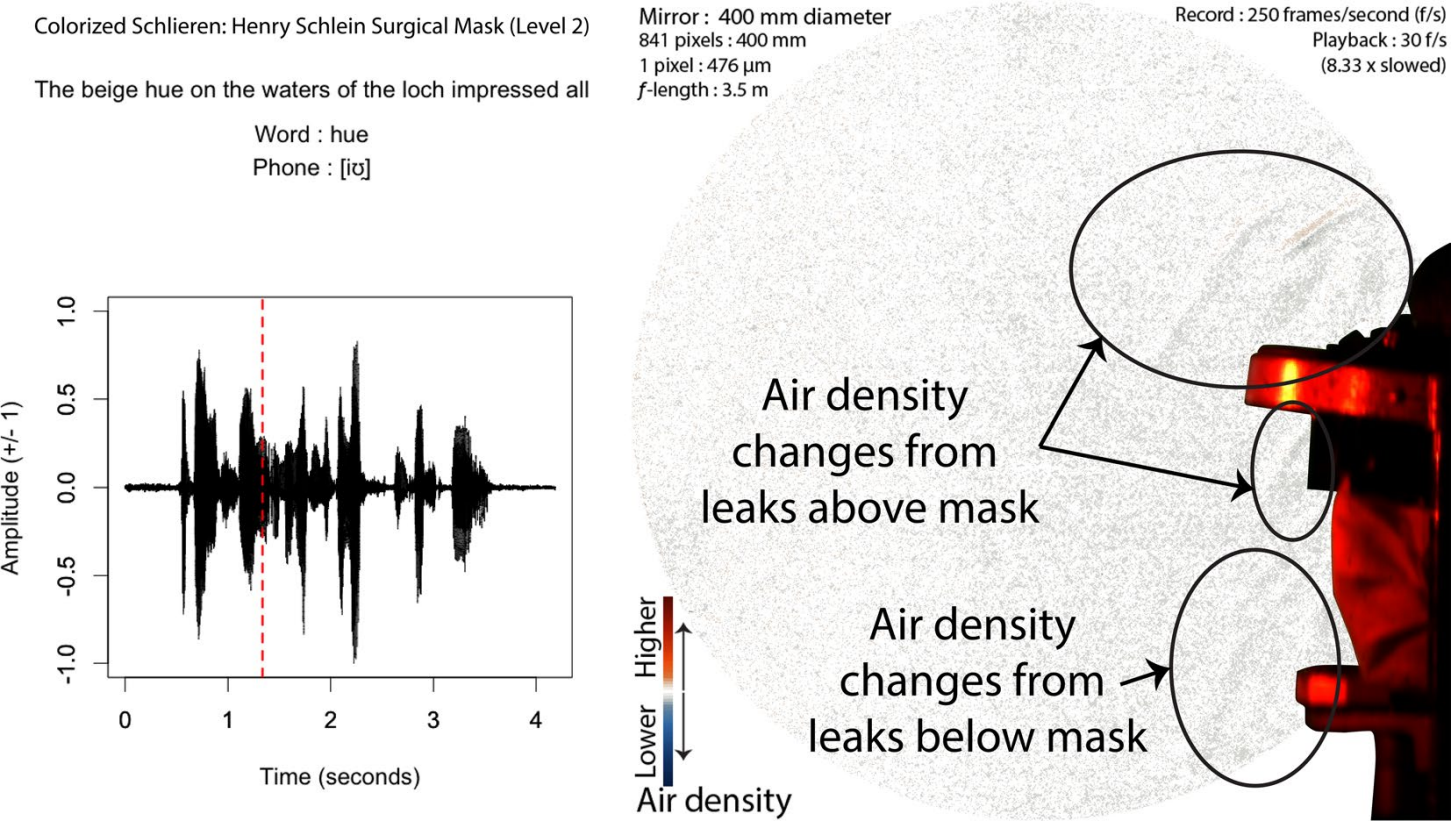
Audio and Schlieren of speech with a tightly fitting surgical mask (Frame 334, 1st block, Henry Schlein surgical mask [level 2]). Air slowly flows out above the eyes, floating out and upward continuously. The red-dashed line in the audio waveform indicates the timing of the schlieren frame.

In contrast, all of the rest of the masks fitted more effectively, slowing down air flow, but allowing leaks from the top and bottom. These leaks produce slow-moving eddies that are difficult to distinguish from the person’s body heat, as seen in Fig. 8. While we cannot see leaks directly from the sides of the mask, the presence of eddies just forward of the mask covering strongly suggests leakage all around the mask, and not just at the top and bottom. Note that this type of result shown in Fig. 8 is representative of every single mask used in this study. Videos of all the masks recordings, as supplied in the supplementary materials S1 referenced at the end of the article, clearly show this type of leakage.

## Discussion

The most important result of this study was to show that all masks, even masks not designed specifically for stopping bacteria or viruses, slow down speech air flow and cause it to penetrate forward much more slowly than speech produced without wearing a face mask. Multi-layered home-made masks and masks designed to stop bacteria and virus slow forward penetration of air more than more porous masks. However, none of the masks completely stop air flow during speech from moving around a speakers’ head. Just as Kellogg uncovered in 1920 with relation to bacterial cultures^3^, there is a trade-off in a mask’s ability to catch material (in this case air), and a tendency for material to leak around the sides of the mask. This means that while masks can slow the spread of air from a speaker, they cannot stop it - regardless of the material from which it is made. The results therefore support the long-standing view that masks can never be as effective as high quality ventilation or outdoor conditions^44^.

Our research is only a small piece of the puzzle that is face-mask efficacy. It does not address how to fit masks more skillfully; proper fitting is one of the most important issues for the effective use of masks. Fit-testing of face masks has been well-studied, but it remains a heuristic process, and research still has not been conducted to show that improving fit reduces the spread viruses such as SARS-CoV-2^45^.

Our research tells us nothing about whether or not particles, including bacteria and viruses, can get through or around the face mask material - it only focuses on large eddy air motion. This means that masks that allow air flow might well block most infectious particles. Even the feeling of air flow from a speaker does not necessarily constitute a risk of infection since that air flow must contain infectious material to cause harm. Similarly, masks that appear to seriously slow air flow might still allow particle through–especially when there are breaks in the seal of the mask that allow unfiltered air to move slowly around the speaker.

There is likely a combination of heat and density contributing to the features seen in the schlieren images, and the method does not discriminate between these. At the very slowest speeds, we have difficulty disambiguating between heat from the speaker’s face and body and heat from the warm air produced during their speech. This means that some of the density changes seen as coming from the top and bottom of the face mask may simply be heat rising from the body of the speaker. Similarly, air flowing from the mask will not show up as the temperature of the air begins to match the environment.

In addition, we did not study the effects of face masks on communication. We do provide acoustic recordings for our readers in our supplementary materials S1 so you can hear that thicker masks muffle speech more than thinner masks. Even more importantly, we have not examined the effects of the loss of visual speech information on speech perception and whether that encourages people to stand more closely to each other while wearing masks. These communication concerns could potentially make a thinner surgical or pollen mask more effective than it would otherwise be as the communication benefits allow for more effective distancing during speech communication. We are currently planning extensive follow up of these questions through ongoing research.

While we do not focus on fitting procedures for face masks, our own experience is that the best-fit masks are large enough for the speakers’ face and have flexible and posable coated metal wire at the nose, like the Temasek silk (9) mask. People who wear glasses will know whether their masks fit reasonably well when their glasses stop fogging up.

Lastly, we did not examine every imaginable type of face mask on the market today - there has been a proliferation beyond count of the types of face masks available. However, our results broadly support the CDC guidelines for mask design as all the masks that were thick, multi-layered, relatively non-porous did a lot to prevent the forward momentum of air flow through the mask.

## Supplementary Information

Supplementary Information.
