# Supplementary material for: Speech air flow with and without face masks
Source: Sci Rep. 2022 Jan 17;12:837. doi: 10.1038/s41598-021-04745-z (PMC8763952; doi:10.1038/s41598-021-04745-z)
Supplement: Supplementary file 1 — Supplementary Information. [file 41598_2021_4745_MOESM1_ESM.docx]

**Supplementary materials**

Supplementary materials include video files of audio, schieren, and face motion for speech without a face mask, two slides from which are shown in Figures 3 and 4. They also include video files of audio and schileren for all of the speech recorded with face masks, slides from which are shown in Figures 5, 6, 7, and 8. Supplementary materials also include the audio files associated with each of these videos, including PRAAT^39^ TextGrids containing labelled and segmented annotations all of the words and segments recorded for this paper. Finally, the supplementary materials contain a README text file describing contents, and a README comma separated file associating base file names with their masks and recorded phrases. Supplementary materials can be found at https://osf.io/rxd3z/?view\_only=9e5379008fec422781d0f49a20257727
